# Tolvaptan in the Treatment of Acute Hyponatremia Associated with Acute Kidney Injury

**DOI:** 10.1155/2013/801575

**Published:** 2013-11-20

**Authors:** Shilpa Gopinath, Kalyana C. Janga, Sheldon Greenberg, Shree K. Sharma

**Affiliations:** ^1^Department of Internal Medicine, Maimonides Medical Center, Brooklyn, NY 11219, USA; ^2^Department of Nephrology, Maimonides Medical Center, 953 49th Street, Brooklyn, NY 11219, USA

## Abstract

Hyponatremia defined as a plasma sodium concentration of less than 135 mmol/L is a very common disorder, occurring in hospitalized patients. Hyponatremia often results from an increase in circulating arginine vasopressin (AVP) levels and/or increased renal sensitivity to AVP, combined with an increased intake of free water. Hyponatremia is subdivided into three groups, depending on clinical history and volume status: hypovolemic, euvolemic, and hypervolemic. Acute symptomatic hyponatremia is usually treated with hypertonic (3%) saline. Syndrome of inappropriate antidiuretic hormone hypersecretion (SIADH) and hypervolemic hyponatremia caused by heart failure or cirrhosis are treated with vasopressin antagonists (vaptans) since they increase plasma sodium (Na^2+^) concentration via their aquaretic effects (augmentation of free-water clearance). The role of tolvaptan in the treatment of acute hyponatremia and conversion of oliguric to nonoliguric phase of acute tubular necrosis has not been previously described.

## 1. Introduction

Acute kidney injury is a frequent complication in critically ill patients and is difficult to manage as it is often accompanied by oliguria or anuria as well as total body fluid overload and edema. Optimal management of volume status as well as normalizing serum sodium levels is essential. Sodium concentration is the major determinant of plasma osmolality; therefore, hyponatremia usually indicates a low plasma osmolality. Low plasma osmolality rather than hyponatremia, per se, is the primary cause of the symptoms of hyponatremia. Hyponatremia not accompanied by hypoosmolality does not cause signs or symptoms and does not require specific treatment [1]. The limitation in the kidney's ability to excrete water in hyponatremic states is, in most cases, due to the persistent action of antidiuretic hormone (ADH, vasopressin). ADH acts at the distal nephron to decrease the renal excretion of water. The action of ADH is, therefore, to concentrate the urine and, as a result, dilute the serum. Under normal circumstances, ADH release is stimulated primarily by hyperosmolality. However, under conditions of severe intravascular volume depletion or hypotension, ADH may be released even in the presence of serum hypoosmolality [1].

Hyponatremia and impaired urinary dilution can be caused by either a primary or a secondary defect in the regulation of AVP secretion or action. The primary forms are generally referred to as the syndrome of inappropriate antidiuresis (SIADH). When osmotic suppression of antidiuresis is impaired for any reason, retention of water and dilution of body fluids occur only if intake exceeds the rate of obligatory and insensible urinary losses. The excess water intake can be due to intravenous administration of hypotonic fluids. In SIADH, the excessive retention of water expands extracellular and intracellular volume, increases glomerular filtration and atrial natriuretic hormone, suppresses plasma renin activity, and increases urinary sodium excretion. This natriuresis reduces total body sodium, and this serves to counteract the extracellular hypervolemia but aggravates the hyponatremia. The osmotically driven increase in intracellular volume results in swelling of brain cells and increases intracranial pressure; this is probably responsible for the symptoms of acute water intoxication. Within a few days, this swelling may be counteracted by inactivation or elimination of intracellular solutes, resulting in the remission of symptoms even though the hyponatremia persists [2]. The management of hyponatremia depends on the severity and duration of symptoms. In a patient with SIADH and few symptoms, the objective is to reduce body water gradually by restricting total fluid intake to less than the sum of urinary and insensible losses. If the symptoms or signs of water intoxication are more severe, the hyponatremia can be corrected by nonpeptide arginine vasopressin (AVP) antagonists that block the antidiuretic effect of AVP. In this paper, the role of tolvaptan in the treatment of acute hyponatremia with acute kidney injury has been described.

## 2. Case Presentation

**Figure 1:**
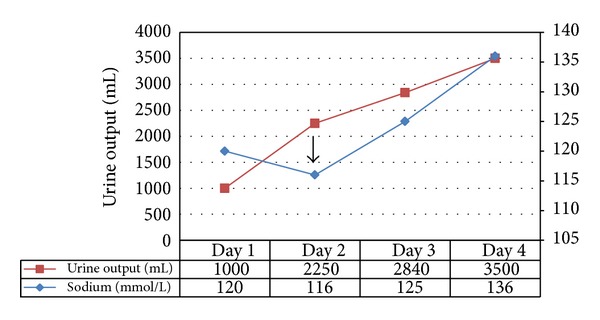
Trend of postoperative urine output and serum sodium levels from postoperative day 1 to day 4 during immediate postoperative period showing acute hyponatremia and acute renal failure followed by immediate recovery on administration of tolvaptan. Arrow represents administration of tolvaptan on Day 2.

A 93-year-old female patient came to the clinic with complaints of haematuria. Her past medical history included hypertension, hypercholesterolemia, depression, osteoporosis, chronic kidney disease stage 3, and morbid obesity. Upon workup she was found to have a polypoid tumor of the urinary bladder with pathologic features of transitional cell carcinoma. She underwent robotic assisted partial cystectomy and normal saline was used for bladder irrigation during the procedure. 24 hours, after partial cystectomy, this patient developed acute oliguric renal failure associated with severe hypotension and she was resuscitated with normal saline boluses. Although the blood pressure returned to normal the patient developed acute hyponatremia with serum sodium levels of 120 mmol/L. Intravenous furosemide 40 mg was administered to induce diuresis. However, there was no response to this. On postoperative day 2 the patient was shifted to the intensive care unit (ICU) with a further drop of serum sodium levels to 116 mmol/L. There was a newly developed right middle lobe pneumonia and signs of pulmonary vascular congestion on chest X-ray. Echocardiography showed a normal ejection fraction and no evidence of pulmonary hypertension. A nephrology consultation was obtained for the management of acute hyponatremia and acute renal failure. Her serum laboratory results further revealed decrease in serum osmolality (247 mosmol/kg; normal levels: 280–301 mosmol/kg) and decreased serum thyroid stimulating hormone (0.5 *μ*IU/mL; normal levels: 0.73–4.60 *μ*IU/mL), whereas serum cortisol levels (27.5 *μ*g/dL; normal 6.7–23.99 *μ*g/dL), B-type natriuretic peptide (1324 pg/mL; normal: 3–82 pg/mL), and serum uric acid (8.8 mg/dL; normal: 2.5–7.5 mg/dL) were elevated. Urine electrolyte results revealed osmolality of 366 mosmol/kg, urine sodium levels were 14 mmol/L, urine potassium was 20.4 mmol/L, chloride levels were 22 mmol/L, creatinine was 48 mg/dL, and fractional excretion of sodium was 0.25%, suggesting prerenal acute renal failure. (Relevant postoperative laboratory data is shown in Table 1.) Due to patient's oliguric state, acute hyponatremia, and congestive heart failure (CHF), tolvaptan 15 mg was given with an intention to convert oliguric to nonoliguric phase of ATN and to treat hyponatremia and hypervolemic state. On postoperative day 3, tolvaptan 15 mg was again administered and 3 hours later polyuria was noted. During postoperative day 4 and day 5, there was a persistent rise of serum sodium levels with return to normal baseline values (the trend of postoperative urine output and serum sodium levels has been graphically represented in Figure 1). Patient was subsequently discharged in 2 days with normal baseline sodium and kidney function.

## 3. Discussion

Hyponatremia is one of the most common electrolyte disorders encountered in clinical practice. Hyponatremia caused by nonosmotic hypersecretion of vasopressin can be divided into hypovolemic, normovolemic, and hypervolemic. Hyponatremia which is not caused by the hypersecretion of vasopressin is seen in cases of pseudohyponatremia, water intoxication, and cerebral salt loss syndrome [3]. Although the pathophysiological process of hyponatremia is complex, AVP is a common etiologic factor. Excess AVP release by osmotic or nonosmotic stimuli or both can lead to sodium and water imbalance. Conventional treatment options for hyponatremia, including water restriction and administration of sodium chloride with or without loop diuretics, do not directly address the underlying water retention induced by excess AVP. Because excessive AVP release is the key etiologic factor in perpetuating the hyponatremia observed in patients with common clinical conditions such as CHF, cirrhosis, nephrosis, and SIADH, therapy that directly antagonizes AVP receptors potentially may offer better outcomes.

Acute hyponatremia is arbitrarily defined as the decrease in serum sodium in a period less than 48 hours [4]. When plasma osmolality decreases rapidly, brain cells gain water and activate protective mechanisms that mitigate against the increase in intracerebral pressure. This involves an increase in interstitial pressure that enhances the movement of extracellular fluid into the cerebrospinal fluid. Failure to adequately undergo this protective process results in cerebral edema. In symptomatic patients, particularly those with risk of complications, acute hyponatremia is a life-threatening medical emergency requiring immediate treatment [4]. The primary goal of therapy in hyponatremia is aimed at achieving a rapid increase in serum sodium to reverse potentially life-threatening cerebral edema. This can be achieved by the administration of hypertonic saline (3% is equivalent to 513 mEq/L sodium) at a dose of 1-2 mL/kg given rapidly and repeated if necessary until symptoms, such as seizures, subside [4]. Hypervolemic hyponatremia should be treated by reduction of water intake and not by increasing the amount of body sodium. Restricting fluid intake to 500 mL per day less than the urinary output is effective but results in a very slow rise of serum sodium levels (1–3 mmol/L per day) and is difficult to maintain, particularly on an ambulatory basis. No absolute level of fluid intake will reduce body water to the same extent in all patients because their size and rate of urine output vary depending on the level of antidiuresis and solute intake. Thus, conventional ways of treating hypervolemic hyponatremia are not optimum, and a better way of promoting water excretion is needed in this condition [5].

Fluid overload is both a consequence and a risk factor for the development of acute kidney injury (AKI) in critically ill patients. Studies in the past have suggested that oliguric AKI carries a higher mortality than nonoliguric AKI [6]. In patients who are fluid overloaded and oliguric, diuretics can be helpful in some cases. While there is no evidence that converting patients from oliguric to nonoliguric renal failure has a beneficial effect on mortality or recovery of renal function after AKI, avoiding fluid overload and inducing a net negative fluid balance correlate with better outcomes after AKI [7]. In the case of oliguric or anuric AKI, diuretics are often utilized to increase the urine output although current evidence suggests that they are best reserved for the treatment of volume overload and hyperkalemia in patients who are likely to respond to them [7].

## 4. Role of Vaptans

Arginine vasopressin (AVP) is a neuropeptide hormone synthesized in the nuclei of the hypothalamus in neuronal cell bodies and released from the posterior pituitary into the bloodstream. AVP has two types of receptors, *V*_1_ and *V*_2_. *V*_1_ receptors are further divided into *V*_1a_ and *V*_1b_. AVP plays a major role in body fluid regulation via *V*_2_ receptors in the renal collecting ducts. When circulating AVP binds to *V*_2_ receptors in the kidneys, reabsorption of free water is increased. This effect is mediated by increased intracellular cyclic adenosine monophosphate (cAMP) and trafficking of aquaporin-2 from intracellular vesicles to the apical plasma membranes [8].

 In the 1990s, a number of nonpeptide vasopressin antagonists were discovered. Tolvaptan (OPC-41061) is one of the potent, highly selective, and orally effective nonpeptide AVP *V*_2_ receptor antagonists developed for the treatment of the hypervolemic and euvolemic forms of hyponatremia. Vaptans are nonpeptide competitive inhibitors of the *V*_2_ receptor located on the basolateral membrane of the collecting ducts principal cells. They bind to the *V*_2_ receptor, preventing the hormone's downstream signaling pathway—the generation of intracellular cAMP and the expression and insertion of aquaporin-2 on the apical membrane. This inhibits water reabsorption and results in the excretion of markedly dilute urine (aquaresis). AVP *V*_2_ receptor antagonists, unlike conventional diuretics, promote free-water excretion without disturbing electrolytic balance and are proven to be clinically useful for the treatment of diseases associated with hyponatremia or fluid retention.

 The study of ascending levels of hyponatremia 1 and 2 (SALT-1 and SALT-2), identically designed, multicenter, randomized, double-blind, placebo-controlled trials, assigned patients with hyponatremia (serum sodium concentration less than 135 mEq/L (mmol/L)) due to SIADH, chronic heart failure, or cirrhosis to oral placebo or oral tolvaptan at a dose of 15 mg daily for up to 30 days. Serum Na was significantly higher within the first 8 h after tolvaptan administration [9]. The effects of tolvaptan were also evaluated in acute and chronic rat models of hyponatremia. A rat model of acutely progressive hyponatremia was induced by infusion of 10 ng/h of DDAVP ([deamino-Cys^1^, D-Arg^8^]-vasopressin, a peptide *V*_2_ agonist) using osmotic minipumps and intragastric water loading. In the acute model, plasma sodium levels were progressively decreased, resulting in severe hyponatremia associated with a high mortality rate (47%). At 1 to 10 mg/kg oral tolvaptan produced dose-dependent aquaresis, leading to an increase in plasma sodium levels. At 3 or 10 mg/kg oral dose of tolvaptan corrected plasma sodium levels and prevented the mortality of animals [10].

## 5. Conclusion

Vaptans have been valuable for the treatment of hypervolemic hyponatremia and in patients with subacute or chronic forms of SIADH. We report a first case known to us where tolvaptan was used in the treatment of hypervolemic hyponatremia and oliguric acute tubular necrosis. Large studies are further needed to prove the efficacy of tolvaptan's role in inducing diuresis and converting oliguric to nonoliguric phase of acute kidney injury and thus reducing morbidity and mortality.

## Supplementary Material

Serum sodium levels and urine output from post operative Day 1 to Day 4.

## Figures and Tables

**Table 1:** Trend of pertinent postoperative clinical and laboratory data.

Laboratory values	Day 1	Day 2	Day 3	Day 4
Sodium (mmol/L)	120	116	125	136
Urine Output (mL)	1000	2250	2840	3500
Hematocrit (%)	33	30.6	27.3	29.7
WBC (×10^9^/L)	18.9	13.9	9.9	11.4
Creatinine (mg/dl)	1.2	1	1.1	1.2
BUN (mg/dl)	30	26	20	18
Potassium (mEq/L)	3.7	4.2	4.2	4.4
