# Supplementary material for: Tolvaptan in the Treatment of Acute Hyponatremia Associated with Acute Kidney Injury
Source: Case Rep Nephrol. 2013 Nov 20;2013:801575. doi: 10.1155/2013/801575 (PMC3914017; doi:10.1155/2013/801575)
Supplement: Supplementary file 1 — Serum sodium levels and urine output from post operative Day 1 to Day 4. [file 801575.f1.docx]

**Serum sodium levels and urine output from post operative Day 1 to Day 4**

A graphic representation of serum sodium levels and urine output during immediate postoperative period showing acute hyponatremia and acute renal failure followed by immediate recovery on administration of tolvaptan. Arrow represents administration of tolvaptan on Day 2.

1. Table 1: Trend of pertinent postoperative clinical and laboratory data

| **Laboratory Values** | **Day 1** | **Day 2** | **Day 3** | **Day 4** |
| --- | --- | --- | --- | --- |
| **Sodium (mmol/L)** | 120 | 116 | 125 | 136 |
| **Urine Output(ml)** | 1000 | 2250 | 2840 | 3500 |
| **Hematocrit (%)** | 33 | 30.6 | 27.3 | 29.7 |
| **WBC(x 10^9^/L)** | 18.9 | 13.9 | 9.9 | 11.4 |
| **Creatinine (mg/dl)** | 1.2 | 1 | 1.1 | 1.2 |
| **BUN(mg/dl)** | 30 | 26 | 20 | 18 |
| **Potassium(mEq/L)** | 3.7 | 4.2 | 4.2 | 4.4 |
